# A Radiotherapy Dose Map-Guided Deep Learning Method for Predicting Pathological Complete Response in Esophageal Cancer Patients after Neoadjuvant Chemoradiotherapy Followed by Surgery

**DOI:** 10.3390/biomedicines11113072

**Published:** 2023-11-16

**Authors:** Wing-Keen Yap, Ing-Tsung Hsiao, Wing-Lake Yap, Tsung-You Tsai, Yi-An Lu, Chan-Keng Yang, Meng-Ting Peng, En-Lin Su, Shih-Chun Cheng

**Affiliations:** 1 Department of Radiation Oncology, Proton and Radiation Therapy Center, Chang Gung Memorial Hospital—Linkou Medical Center, 5 Fu-Shin Street, Kwei-Shan, Taoyuan 333, Taiwan; 2 Department of Medical Imaging and Radiological Sciences, Healthy Aging Research Center, Chang Gung University, Taoyuan 333, Taiwan; 3 Department of Post Baccalaureate Medicine, Kaohsiung Medical University, Kaohsiung 807, Taiwan; 4 Department of Otolaryngology—Head and Neck Surgery, Chang Gung Memorial Hospital—Linkou Medical Center, 5 Fu-Shin Street, Kwei-Shan, Taoyuan 333, Taiwan; 5 Division of Hematology and Oncology, Department of Internal Medicine, Chang Gung Memorial Hospital at Linkou Branch, Chang Gung University College of Medicine, Taoyuan 333, Taiwan; 6 Department of School of Medicine, National Yang Ming Chiao Tung University, Taipei 112, Taiwan; 7 Taiwan AI Labs, Taipei 103, Taiwan

**Keywords:** deep learning, pathological complete response, esophageal cancer, neoadjuvant chemoradiotherapy, artificial intelligence, radiotherapy treatment planning, radiomics, dosiomics

## Abstract

**Simple Summary:**

The authors conducted this research to improve the prediction of how patients with esophageal cancer respond to a treatment called neoadjuvant chemoradiotherapy, which can enhance survival rates. However, doctors struggle to accurately predict how well a patient will respond to this treatment using existing imaging methods. To address this, the researchers developed a computer-based method called DCRNet, that not only analyzes medical images but also considers the distribution of radiation therapy doses on the radiotherapy treatment plans to make more accurate predictions. They tested this method on 80 patients with esophageal cancer and found that the HRNetV2p model with DCR performed the best, significantly improving prediction accuracy compared to other models. This breakthrough has the potential to help doctors better anticipate patient responses to treatment, which could lead to more personalized and effective care, and improving the treatment planning of radiotherapy.

**Abstract:**

Esophageal cancer is a deadly disease, and neoadjuvant chemoradiotherapy can improve patient survival, particularly for patients achieving a pathological complete response (ypCR). However, existing imaging methods struggle to accurately predict ypCR. This study explores computer-aided detection methods, considering both imaging data and radiotherapy dose variations to enhance prediction accuracy. It involved patients with node-positive esophageal squamous cell carcinoma undergoing neoadjuvant chemoradiotherapy and surgery, with data collected from 2014 to 2017, randomly split into five subsets for 5-fold cross-validation. The algorithm DCRNet, an advanced version of OCRNet, integrates RT dose distribution into dose contextual representations (DCR), combining dose and pixel representation with ten soft regions. Among the 80 enrolled patients (mean age 55.68 years, primarily male, with stage III disease and middle-part lesions), the ypCR rate was 28.75%, showing no significant demographic or disease differences between the ypCR and non-ypCR groups. Among the three summarization methods, the maximum value across the CTV method produced the best results with an AUC of 0.928. The HRNetV2p model with DCR performed the best among the four backbone models tested, with an AUC of 0.928 (95% CI, 0.884–0.972) based on 5-fold cross-validation, showing significant improvement compared to other models. This underscores DCR-equipped models’ superior AUC outcomes. The study highlights the potential of dose-guided deep learning in ypCR prediction, necessitating larger, multicenter studies to validate the results.

## 1. Introduction

Esophageal cancer presents a grim prognosis with a 5-year survival rate below 25% [1]. Particularly prevalent in Asia, esophageal squamous cell carcinoma (ESCC) predominates over other forms, defying the Western trend where adenocarcinoma predominates [2]. Despite surgical advancements, disease recurrence remains a pressing concern, prompting exploration into neoadjuvant chemoradiotherapy (nCRT) as a means to bolster long-term survival in locally advanced ESCC patients [3,4]. Recent meta-analysis results indicate that at least 24–32% of patients attain a pathological complete response (ypCR) following nCRT, setting the stage for a “watch-and-wait” strategy as an alternative to surgery, aimed at reducing adverse events [5]. Regrettably, present imaging techniques, including computed tomography, positron emission tomography, endoscopic ultrasound, and magnetic resonance imaging, suffer from inadequate sensitivity (0.35, 0.62, 0.01, and 0.80, respectively) in detecting ypCR [5]. Consequently, there is a growing interest in computer-aided detection methods, like radiomics and machine learning, to facilitate ypCR prediction, aiding both patients and physicians in treatment planning.

While prior research has delved into various machine learning models to predict treatment responses in esophageal cancer centering on imaging features [6,7,8,9,10,11,12], these studies have chiefly focused on imaging data, overlooking the potential ramifications of variations in radiotherapy dose planning practices on treatment responses. Notably, previous published studies have demonstrated that a good response after chemoradiotherapy, particularly a ypCR is prognosticated for excellent overall survival and recurrence-free survival in ESCC patients [13,14,15]. Thus, the capability to predict a ypCR after nCRT in individual patients remains a crucial yet unmet need.

Our study introduces an innovative approach to predicting esophageal cancer patient responses to nCRT. We employ a deep learning methodology that transcends conventional reliance on imaging data alone, incorporating the variances in radiotherapy dose mapping. Specifically, we propose a radiotherapy dose map-guided deep learning model that predicts a ypCR after nCRT in patients with ESCC.

## 2. Materials and Methods

### 2.1. Patients

This study was conducted with the approval of the institutional review boards at Chang Gung Memorial Hospital. To safeguard patient privacy, the data used were de-identified, obviating the need for informed consent. The study adhered to the Standards for Reporting of Diagnostic Accuracy (STARD) guidelines.

**Figure 1:**
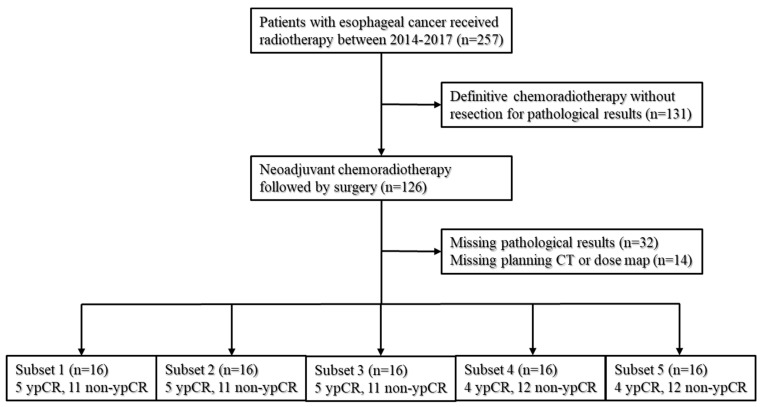
Patient inclusion and grouping.

The data employed in this research were derived from patients diagnosed with node-positive ESCC who underwent nCRT followed by surgery from January 2014 to December 2017. To ensure robustness, the patient cohort was randomly divided into five subsets for 5-fold cross-validation, with each subset maintaining an equivalent ratio of patients who achieved a ypCR and those who did not, as illustrated in Figure 1.

All patients in the study received platinum-based chemotherapy concurrently with radiotherapy, with the total radiation dose ranging from 41.4 to 50.4 Gy. The gross tumor volume (GTV) encompassed both the primary tumor and the involved regional nodes, as identified through endoscopy, CT, or PET/CT scans. The clinical target volume (CTV) extended 3–4 cm along the esophagogastric wall, both proximally and distally to the GTV, with an added 1 cm radial expansion to accommodate potential microscopic periesophageal spread.

Both planning CT scans and radiation dose maps were obtained for the development of the model. Following nCRT, enrolled patients underwent radical esophagectomy within a window of 4 to 8 weeks. Specialized pathologists, with expertise in ESCC, assessed the pathological response. The criteria for a ypCR were met when no viable cancer cells were observed in the primary tumor and regional lymph nodes.

### 2.2. Development of the Algorithm

**Figure 2:**
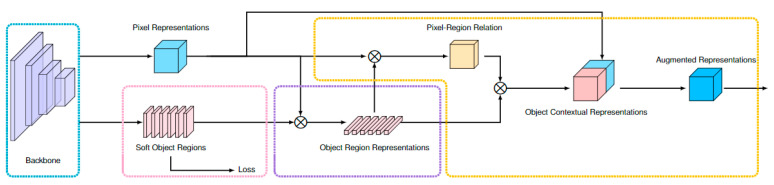
The architecture of DCRNet.

The ypCR prediction algorithm represents an advancement of the state-of-the-art OCRNet (object contextual representations network) developed by Yuan et al. [16]. This algorithm is specifically designed for semantic segmentation tasks employing convolutional neural networks. To enhance its performance, the ypCR algorithm introduces the concept of soft regions, which prove particularly useful in approximate auto-planning scenarios. By integrating information related to radiation therapy dose distribution into the contextual representations of regions, the ypCR algorithm aims to refine the accuracy of semantic segmentation, as depicted in Figure 2. In this algorithm, object regions are defined based on varying levels of radiation isodose. To distinguish it from OCRNet, this variant is referred to as DCRNet (dose contextual representations network). The improved performance and distinctive attributes of the ypCR algorithm render it a valuable asset in the realms of semantic segmentation and radiation therapy planning.

**Figure 3:**
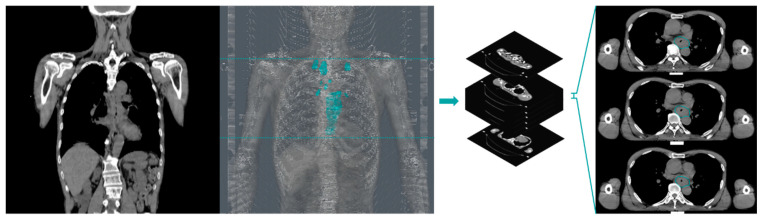
Image preprocessing.

DCRNet is constructed upon HRNetV2p [17], which was initially pre-trained on the Cityscapes dataset, serving as the foundational framework for extracting feature maps from planning CT images in a 2.5D fashion. The algorithm takes CT data as input, incorporating three consecutive slices covering the CTV from the radiotherapy (RT) structure set into the backbone model, as illustrated in Figure 3. Uniform ground-truth labels are assigned to all input units of the same patient, according to the patient’s pathological response.

To produce dose contextual representations, we employed channel softmax normalization for 10 soft regions, each corresponding to a 10% radiation isodose level (ranging from 0% to 100%), akin to dose-volume histogram analysis. In the model training phase, we utilized the dice loss function to supervise the model, simulating conditions akin to rough auto-planning. Subsequently, the resulting dose representation was amalgamated with the pixel representation to establish the dose contextual representations, following a methodology consistent with prior works [16].

**Figure 4:**
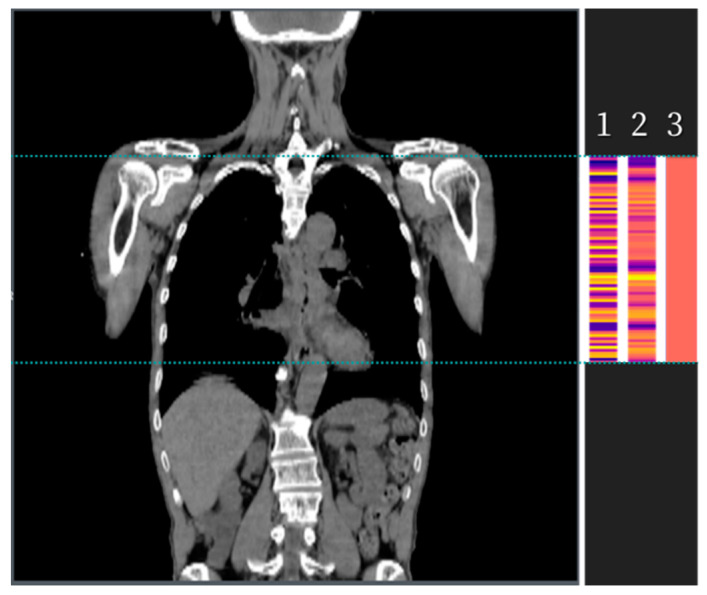
Three methods to summarize possibility in patient: (1) the maximum value across the CTV, (2) the maximum between mean values of every three continuous slices, (3) the mean value across the CTV.

To reflect the pathological outcomes, a fully connected layer was incorporated, consisting of two nodes. The output layer, comprising a single node, was supervised by a cross-entropy loss function designed to predict the likelihood of residual disease presence on each CT slice. The results derived from all input CT images for a given patient were summarized in three distinct manners: (1) the maximum value across the CTV, (2) the maximum between mean values obtained from every three consecutive slices, and (3) the mean value across the CTV (Figure 4). Performance comparisons were conducted across different models and summarization methods.

For training, we employed an Adam optimizer with a learning rate set at 1 × 10^−4^, implementing gradient clipping. To enhance training focus on challenging instances, we utilized Online Hard Example Mining (OHEM) [18]. Model training took place on a system equipped with an Intel Xeon E5-2630 v4 2.20 GHz CPU, an NVIDIA Tesla T4 GPU boasting 16 GB of memory, and 128GB of RAM. The algorithm was implemented in PyTorch, building upon the OCRNet codebase.

### 2.3. Statistical Analysis

Categorical variables underwent statistical comparison using the χ^2^ or Fisher exact test, while numeric variables were assessed using the Kruskal–Wallis test. The performance of DCRNet models was gauged using the area under the ROC curve (AUC) through 5-fold cross-validation, with *p* values for differences determined through DeLong’s test [19]. Cutoff points for accuracy, sensitivity, and specificity were established using the Youden Index. Calibration performance and clinical utility were evaluated via calibration curves and decision curves. Statistical significance was denoted by a 2-tailed *p* value less than 0.05. Data visualization was accomplished using Python software version 3.8, while R software version 4.0.5 was employed for statistical analysis.

### 2.4. Five-Fold Cross-Validation

Five-fold cross-validation is a widely used technique in machine learning for model evaluation and performance estimation. It involves the division of a dataset into five equal subsets or “folds”. The model is then trained and tested five times, where in each iteration, one fold is designated as the test set, and the remaining four folds are used for training. This process is repeated five times, ensuring that each fold is used as a test set exactly once. The results from these iterations are then averaged to provide a comprehensive and reliable assessment of the model’s performance. Five-fold cross-validation helps reduce the impact of randomness and ensures that the model’s performance estimate is more representative of its generalization capabilities compared to a single train/test split, making it a valuable tool in model selection and performance assessment.

## 3. Results

### 3.1. Baseline Characteristics

In this study, 80 patients participated, with a mean age of 55.68 (±9.5) years, consisting of 76 (95%) males and four (5%) females. Predominantly, the lesions were situated in the middle part (41.1%), and the majority (71.2%) were diagnosed with stage III disease. The observed ypCR rate across all patients stood at 28.75%, aligning with findings from previous research. Notably, there were no statistically significant differences in age, lesion location, clinical staging, radiation dosage, or overall survival outcomes between the ypCR and non-ypCR groups (Table 1).

### 3.2. The Comparison of Three Methods in the Patient Summary

**Figure 5:**
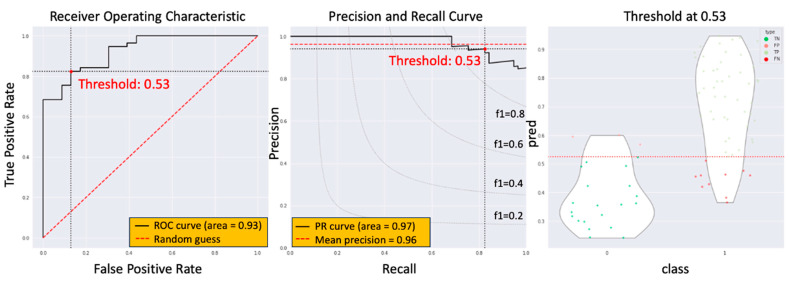
Model performance of the first summary method.

The model computed a potential non-ypCR value based on images. To consolidate this value effectively across all images for a given patient, we assessed three distinct summarization methods. Initially, we adopted the approach of selecting the maximum value within the CTV. Subsequently, through thorough evaluation, we identified an optimal cutoff for this potential value at 0.525. This particular method yielded an impressive AUC score of 0.928, accompanied by a sensitivity of 0.845 and specificity of 0.852, as illustrated in Figure 5.

**Figure 6:**
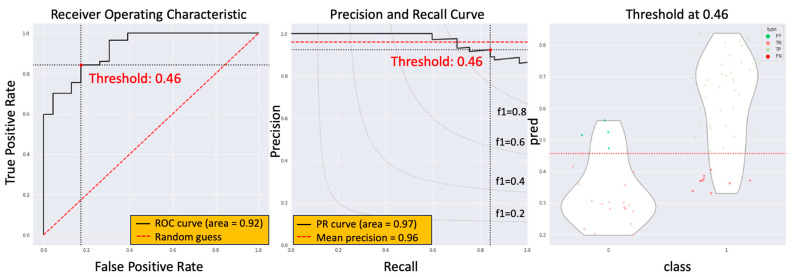
The model performance of the second summary method.

We employed the second method, which involved identifying the maximum among the mean values of every three consecutive slices. Through optimization, a possibility value cutoff of 0.457 was determined as optimal. This method yielded an AUC of 0.923, alongside a sensitivity of 0.840 and a specificity of 0.826, as depicted in Figure 6.

**Figure 7:**
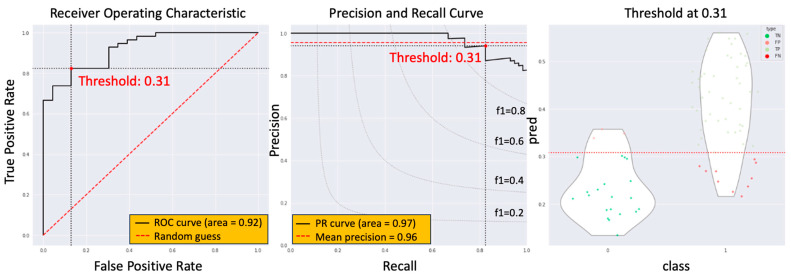
The model performance of the third summary method.

The third approach involved computing the mean value across the CTV, with an optimal cutoff set at 0.309. This method yielded an AUC of 0.924, a sensitivity of 0.825, and a specificity of 0.869 (Figure 7). These findings highlight the similarity in predictive performance among all three methods for identifying non-ypCR cases, albeit with minor discrepancies in optimal cutoff values and performance metrics.

### 3.3. The Model Performance of DCRNet in Pathological Results

We observed no significant performance differences among the three summary methods and opted for simplicity by selecting the maximum value across the CTV. Subsequently, we conducted a comparative analysis of four backbone models (HRNetV2p, ResNet-101, EfficientNet, and DenseNet-121), both with and without dose contextual representations (Table 2). The HRNetV2p model integrated with dose contextual representations (DCR) outperformed the others, yielding an AUC of 0.928 (95% CI, 0.884–0.972) through 5-fold cross-validation, demonstrating a notable superiority over alternative models. Models incorporating DCR exhibited significantly better AUC outcomes in comparison to those without DCR.

## 4. Discussion

Accurately detecting ypCR in esophageal cancer remains challenging with traditional imaging methods, such as computed tomography, positron emission tomography, endoscopic ultrasound, and magnetic resonance imaging, as their sensitivity ranges from 0.35 to 0.80 [5]. To overcome this limitation, computer-aided detection techniques, including radiomics and machine learning, have garnered interest as tools to enhance ypCR prediction. Prior research by Zhang et al. utilized a support vector machine model, achieving high accuracy by combining clinical, conventional PET, and radiomic PET features [6]. Beukinga et al. [7] and Yang et al. [8] developed multivariate logistic regression models incorporating textural features from PET and CT scans. Ypsilantis et al. demonstrated that a convolutional neural network outperformed traditional machine learning methods, such as support vector machine, logistic regression, random forest, and gradient boosting [9]. Hou et al. conducted two studies comparing an artificial neural network and support vector machine based on CT and MRI features, both yielding accurate treatment response predictions [10,11]. Notably, no significant difference in predictive performance emerged between artificial neural network and support vector machine models, emphasizing the model choice may not be the primary factor [10,11]. Additionally, recent research delves into radiomic features of the peritumoral region, deemed reflective of the tumor microenvironment. Hu et al. [12] developed a support vector machine model with a radial basis function kernel, enhancing the AUC by integrating intratumoral and peritumoral features. While these studies underscore the efficacy of imaging features and machine learning models in predicting treatment response in esophageal cancer, wider clinical adoption awaits further validation in larger patient cohorts and the standardization of feature extraction methods.

The current study introduces an innovative approach, the radiotherapy dose map-guided deep learning method, to predict esophageal cancer patients’ responses to nCRT. This method integrates imaging data with radiotherapy dose map variations, offering a more comprehensive assessment of patient responses. Notably, the study population’s demographics and ypCR rate aligned with previous research findings [5]. There were no statistically significant differences observed in age, tumor location, clinical stage, or radiation dose between the ypCR and non-ypCR groups. Three methods were employed to summarize the potential value of non-ypCR predicted by the model. The first method, taking the maximum value across the CTV, yielded an AUC score of 0.928, a sensitivity of 0.845, and a specificity of 0.852, with an optimal cutoff at 0.525. The second method, based on the maximum between the mean values of three consecutive slices, produced an AUC of 0.923, a sensitivity of 0.840, and a specificity of 0.826, with an optimal cutoff at 0.457. The third method, utilizing the mean value across the CTV, resulted in an AUC of 0.924, a sensitivity of 0.825, and a specificity of 0.869 with an optimal cutoff at 0.309. These findings indicate that all three methods effectively predicted the presence of non-ypCR, albeit with variations in optimal cutoff values and performance metrics. To assess the impact of dose map information, this study compared four distinct deep learning models (HRNetV2p, ResNet-101, EfficientNet, and DenseNet-121) both with and without dose contextual representations. The simplest summary method, the maximum value across the CTV, was chosen. Results revealed that the HRNetV2p model, when enhanced with dose contextual representations (DCR), outperformed others, achieving an AUC of 0.928 through 5-fold cross-validation. This outcome marked a significant improvement over models lacking DCR. Notably, models without DCR demonstrated AUC results in line with prior research findings [6,7,8,9,10,11,12].

Given the small number size of this study, five-fold cross-validation method was performed for model evaluation to address the limitations of the traditional train/test split method. The comprehensive process of 5-fold cross-validation provides several key advantages compared to a single train/test split. First, it delivers a more reliable performance estimate by averaging results over five iterations, reducing the influence of random data splits and outliers. Second, it maximizes data utilization, as all data points are used for both training and testing, enhancing the model’s ability to learn from the entire dataset. Third, it mitigates the risk of overfitting by testing the model on five different subsets, making it less likely to memorize peculiarities of a single split. Moreover, it assesses model robustness by revealing whether the model’s performance is consistent across the folds, indicating its generalization capabilities. Lastly, five-fold cross-validation enables fair model comparisons, aiding in the selection of the best-performing model, making it a valuable tool in machine learning for accurate and reliable model assessment.

In our study, a noteworthy observation was the statistically significant difference in the male-to-female ratio between the ypCR group and the non-ypCR group, with the ypCR group having a higher proportion of female patients. This finding aligns with a prior study that identified female sex as an independent favorable prognostic factor in patients with ESCC who underwent definitive radiotherapy [20]. It is worth noting that our study cohort consisted of 95% male patients, reflecting the male-to-female ratio observed in the general population of individuals diagnosed with ESCC in Taiwan [21]. According to the Cancer Registry Annual Report 2020 published by the Ministry of Health and Welfare of Taiwan in December 2022, out of the 2652 newly diagnosed ESCC cases in 2020, 93% were male patients [21]. However, it is essential to recognize that while ESCC is typically more prevalent in males, with a global male-to-female age-standardized incidence rate ratio (ASIR) of approximately 3.3:1, significant variations exist by country [22]. For instance, in the Republic of Korea and several Baltic/European countries, including Belarus, Ukraine, Slovakia, Lithuania, Latvia, and Estonia, studies have reported extremely high male-to-female ASIRs, exceeding 10:1 and even reaching as high as 21:1 for ESCC [22]. The male-to-female ASIR for ESCC in Taiwan is similar to these countries, highlighting the geographical diversity in the incidence of this disease.

This study highlights the potential of a radiotherapy dose map-guided deep learning approach, combining imaging data and radiotherapy dose map variables, to predict ypCR effectively. These results hold the promise of enhancing patient response prediction, enabling more personalized and effective care. This includes avoiding unnecessary surgeries and optimizing radiotherapy treatment planning by incorporating the algorithm into the treatment planning system, thereby potentially improving patient outcomes and treatment strategies. Nevertheless, several limitations in the experiment may affect the validity of the results. Firstly, the relatively small sample size, comprising only 80 patients, could potentially restrict the generalizability of the findings, as larger sample sizes are often required for more robust and accurate predictions. Secondly, the use of data from a single center introduces potential bias into the results, particularly if there are variations in patient populations or treatment protocols across centers. Finally, the study did not evaluate the model’s impact on patient outcomes, including survival and quality of life, which should be addressed in future research to provide a more comprehensive understanding of its clinical significance.

## 5. Conclusions

This study introduced a novel approach to predict nCRT response in esophageal cancer patients, employing a deep learning method that considers both imaging data and radiotherapy dose map variations. The HRNetV2p with DCR model demonstrated superior performance, achieving an AUC of 0.928 through 5-fold cross-validation, signifying significant improvement over alternative models. While all three summary methods yielded similar results for non-ypCR prediction, the simplicity of selecting the maximum value across the CTV was preferred. This research highlights the promise of radiotherapy dose map-guided deep learning in enhancing ypCR prediction. However, further studies involving larger patient cohorts are essential to validate these findings and unlock the full potential of the proposed methodology.

## Figures and Tables

**Table 1:** Patients’ characteristics.

Characteristics	Category	All Patients, *n* (%)	ypCR, *n* (%)	Non-ypCR, *n* (%)	*p*
Sex	Male	76 (95)	20 (87.0)	56 (98.2)	0.04
	Female	4 (5)	3 (13.0)	1 (1.8)	
Age (year)	Mean (SD)	55.68 ± 9.5	55.48 ± 7.3	55.76 ± 10.3	0.28
Location	Proximal	5 (8.9)	3 (13.1)	2 (6.1)	0.16
	Middle	23 (41.1)	12 (52.2)	11 (33.3)	
	Distal	28 (50)	8 (34.8)	20 (60.6)	
Clinical stage	II	5 (6.3)	2 (8.7)	3 (5.3)	0.70
	III	57 (71.2)	17 (73.9)	40 (70.1)	
	IVA	18 (22.5)	4 (17.4)	14 (24.6)	
Total radiation dose (Gy)		44.0 ± 2.1	44.5 ± 2.4	43.8 ± 2.8	0.81
OS (month)		24.1 ± 13.7	27.7 ± 16.2	22.6 ± 14.8	0.08

SD, standard deviation. OS, overall survival.

**Table 2:** Comparison of predictive AUC in 5-fold cross validation.

AUC (95% CI)	ypCR	ypT0	ypN0
HRN + DCR	0.928 (0.884–0.972)	0.939 (0.928–0.950)	0.891 (0.881–0.901)
HRN	0.865 (0.856–0.873)	0.877 (0.865–0.889)	0.859 (0.846–0.872)
RN + DCR	0.829 (0.809–0.849)	0.845 (0.828–0.862)	0.813 (0.799–0.827)
RN	0.763 (0.751–0.775)	0.775 (0.759–0.791)	0.751 (0.733–0.769)
EN + DCR	0.836 (0.818–0.854)	0.847 (0.825–0.869)	0.825 (0.806–0.844)
EN	0.766 (0.749–0.783)	0.780 (0.756–0.804)	0.752 (0.736–0.768)
DN + DCR	0.832 (0.812–0.852)	0.844 (0.826–0.862)	0.820 (0.803–0.837)
DN	0.761 (0.742–0.780)	0.776 (0.756–0.796)	0.746 (0.731–0.761)

## Data Availability

Data supporting reported results can be provided upon request.
